# Supplementary material for: Convergent evolution in a large cross-cultural database of musical scales
Source: PLoS One. 2023 Dec 13;18(12):e0284851. doi: 10.1371/journal.pone.0284851 (PMC10718441; doi:10.1371/journal.pone.0284851)
Supplement: S1 File — (PDF) [file pone.0284851.s001.pdf]

Supporting information for "Convergent evolution in a large cross-cultural database of musical scales"

John M. McBride[1,*], Sam Passmore[2,3], and Tsvi Tlusty[1,4,*]

[1]Center for Soft and Living Matter, Institute for Basic Science, Ulsan 44919, South Korea
[2]Faculty of Environment and Information Studies, Keio University, Fujisawa, Japan
[3]Evolution of Cultural Diversity Initiative, College of Asia and the Pacific, Australian National University
[4]Departments of Physics and Chemistry, Ulsan National Institute of Science and Technology, Ulsan 44919, South Korea
[*]jmmcbride@protonmail.com, tsvitlusty@gmail.com

November 22, 2023

S1 Appendix. Inferring octave scales. The database exists in two forms: the raw data (382 theory scales, and 434 measured scales), and a set of octave scales that is generated procedurally from the raw data according to five choices. For a complete workflow from source to database, including examples, see Fig. 1 and Fig. 2. The five choices are:

(i) Theory scale intonation: Theory scales are typically written in symbolic notation (*e.g.*, solfège, swara), which can then be converted to numerical sequences by matching the symbols to a tuning system (*e.g.*, 12-tone equal temperament, 12-TET). For each theory scale in the database, we provide a set of tuning schemes that were plausibly used, so a single theory scale may have several versions in different tunings.

(ii) Theory scale variants: The tonic is the first note in all theory scales. However, one may also ignore tonality and include all variants from all theory scales by taking different notes as starting points, thereby forming circular permutations of the original scale. This would create many duplicates since theory scales tend to contain many scales that are variants of each other.

(iii) Measured scale variants: Tonality is rarely clear in sources of measured scales, and therefore it is impossible to uniquely identify the order of notes as intended by the performer. For these scales, one can: (i) include only those where the tonic is known; (ii) include all plausible scales (all variants); (iii) or include more than one scale if there are multiple octaves, without including variants. We define a plausible scale on an instrument tuning as a sequence of notes that span an octave.

(iv) Measured scale octave error tolerance: In practice, measured intervals exhibit random fluctuations, so we accept scales if the intervals sum to $1200 \pm O$ cents, where O is the deviation tolerance.

(v) Measured scale missing octave addition: In some cases, sources indicated that the octave was used in performances, but reported the scales without the octave. For these cases, we can choose to include the missing interval that is needed to complete the octave (affects 41 out of 434 samples). This happens when, for example, measured scales inferred from recordings are collapsed onto a single octave; in this case, we assume that the final interval was omitted simply because it is redundant information.

Different sets of choices introduce variations in the database. In the main manuscript we report statistics for the database created according to the following choices: (i) We match theory scales from each musical tradition to a set of tuning systems given in SI Table 2. (ii) We do not include all possible variants of theory scales. (iii) We do not exclude scales inferred from instrument tunings if we do not know the tonic, but we do not include all possible variants. (iv) We use a tolerance of $O = 50$, noting that 73 % of extracted scales are within 10 cents of the octave (Fig. 3). (v) We include scales that require an extra interval to complete the octave. In total, this results in 896 scales (434 theory scales, 384 scales from instrument tunings, and 78 scales from song recordings). The theory scales span 6 regions, while the measured scales span 8 regions, and 46 countries (main manuscript Fig. 2).

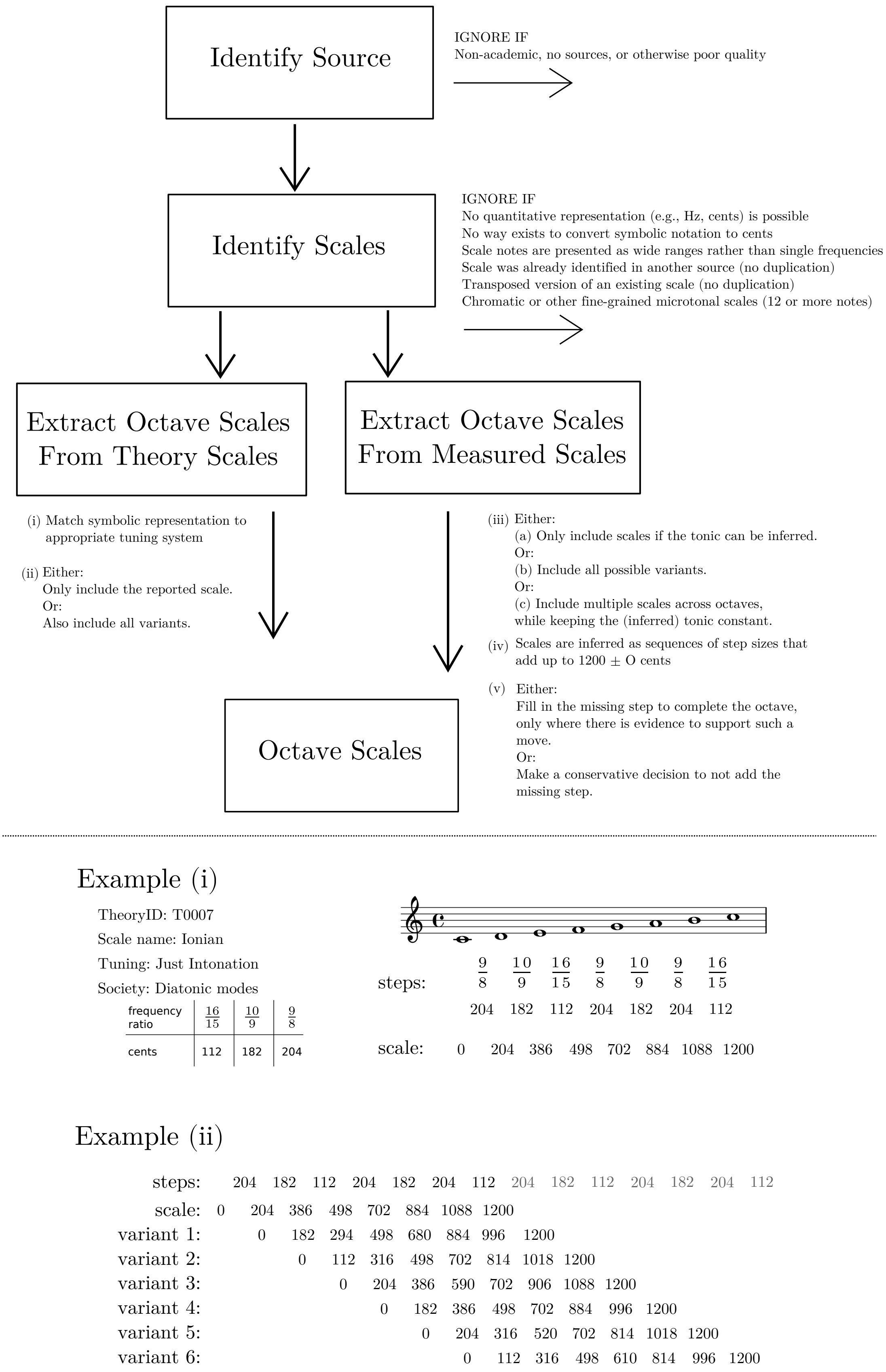

Figure 1: Top: Schematic for construction of scale database. Appropriate sources are first identified, and out of these we identify scales that have unambiguous quantitative representations. This results in a set of 'theory scales', and a set of 'measured scales'. From these two sets, we create a set of 'octave scales', according to a set of five (i – v) choices. Bottom: Examples of how choices (i) and (ii) are implemented. (i) Most theory scales are represented symbolically, and we require some kind of code to convert the symbolic representation into a quantitative representation. (ii) For purposes of analysis, if one wants to ignore the concept of tonality (here referring to the idea that positions in a scale are not equivalent, and that scales start at the tonic), one can include all possible variants that start on different positions.
...

Example (iii)

MeasID: M0131

Instrument: Xylophone

Methodology: Stroboconn used along with recordings of instrument notes

Society: Malinké

Example (a)

Tonic: no info

Do not include any scales

Example (b)

steps:		174	182	177	159	174	168	169	163	177	162	183	175	163	170	180	162	193
scale:	0	174	356	533	692	866	1034	1203										
variant 1:		0	182	359	518	692	860	1029	1192									
variant 2:			0	177	336	510	678	847	1010	1087								
variant 3:				0	159	333	501	670	833	1011	1172							
variant 4:					0	174	342	511	674	851	1013	1196						
variant 5:						0	168	337	500	677	839	1022	1197					
variant 6:							0	169	332	509	671	854	1029	1192				
variant 7:								0	163	340	502	685	860	1023	1193			
variant 8:									0	177	339	522	697	860	1030	1210		
variant 9:										0	162	345	520	683	853	1033	1195	
variant 10:											0	183	358	521	691	871	1033	1226
variant 11:																		

Example (c)

steps:		174	182	177	159	174	168	169	163	177	162	183	175	163	170	180	162	193
scale:	0	174	356	533	692	866	1034	1203										
variant 7:								0	163	340	502	685	860	1023	1193			

Example (v)

MeasID: M0136

Scale name: Bac

Instrument: Dan Tranh

Methodology: Computational analysis of recording of instrument notes

Society: Vietnamese

explicit steps:		189	312	197	193	
implicit steps:						309
scale:	0	189	501	698	891	1200

Figure 2: continuing from Fig. 1...
(iii) sources that contain 'measured scales' rarely explicitly include detailed information about tonality, let alone which note may be considered a starting note ('tonic'). Thus, one can choose to: (a) Only include scales if there is evidence for which note(s) the performer considers a tonic. (b) Include all possible variants where the final note sums to 1200 ± O cents. (c) Identify one potential 'tonic', and only include scales that start on that note, or on notes that are related to the 'tonic' by an octave relation. (iv) The value of O needs to be specified; we choose O = 50 cents. (v) Some sources indicate that a full octave is used, and for reasons of concision they do not report the final interval leading to the octave. In this case, one can choose to include the final interval (shown in red) or not.

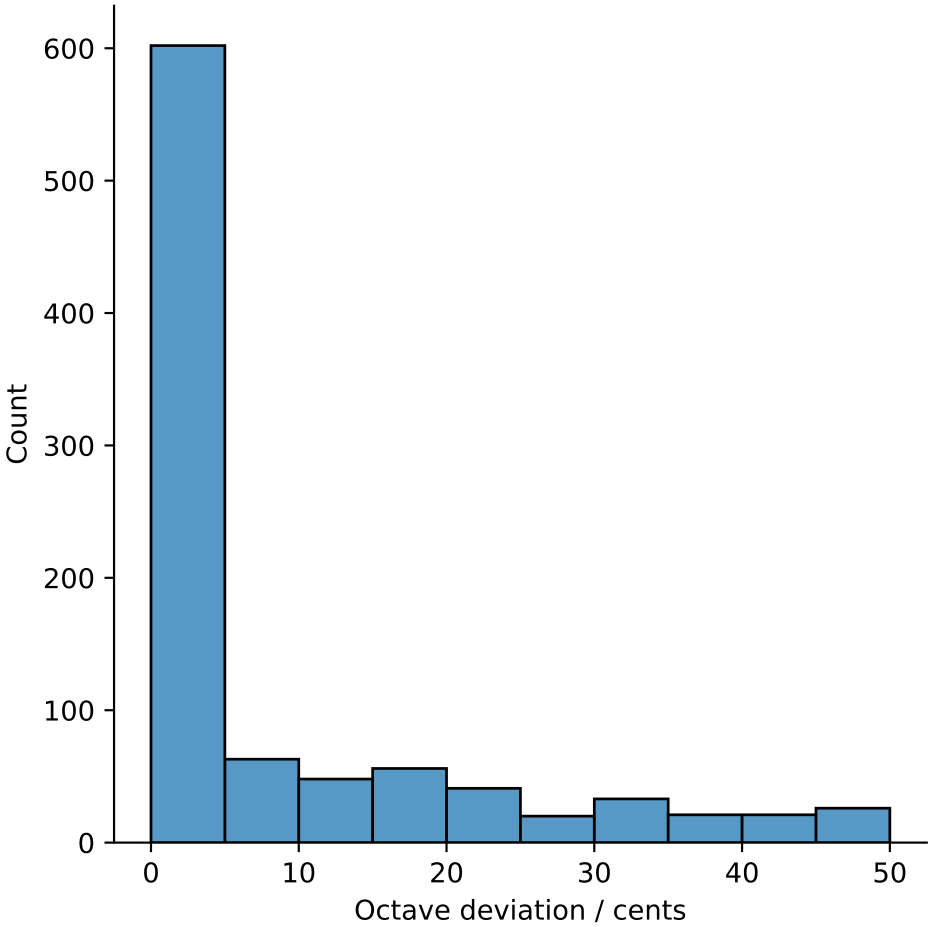

Figure 3: Distribution of deviations of final notes from the octave for all octave scales ($O = 50$).

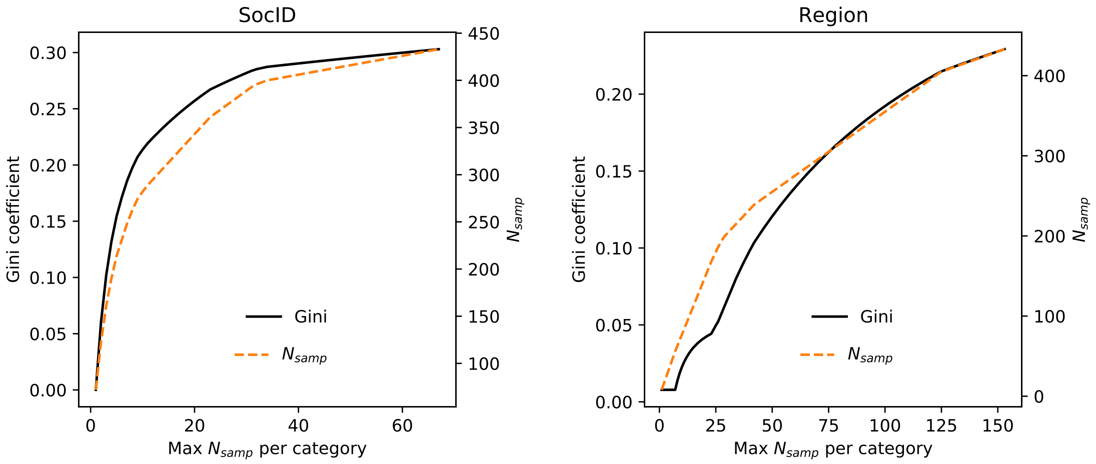

Figure 4: Total number of scales N_{samp}, and Gini coefficient as a function of the maximum number of scales allowed per category: SocID, left; Region, right.

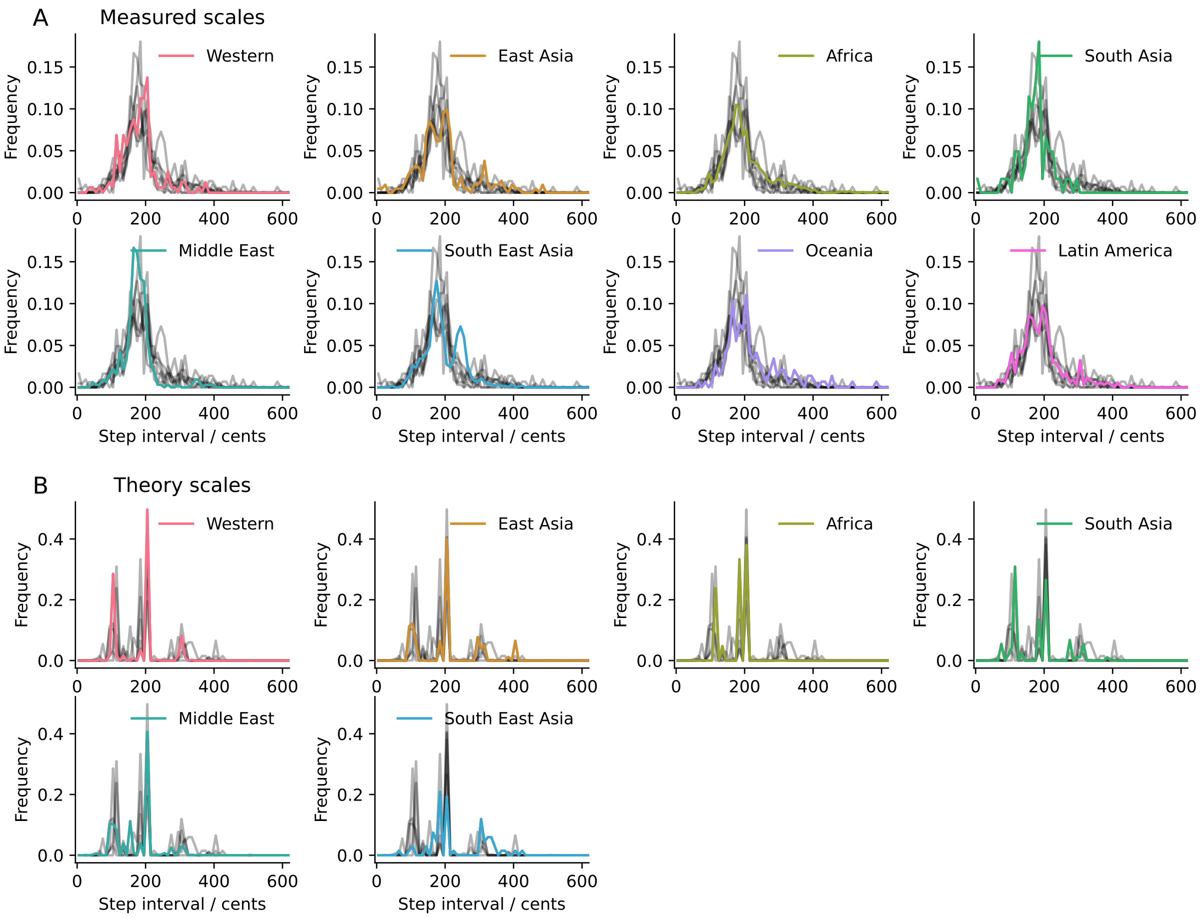

Figure 5: Step interval distributions for Measured scales (A) and Theory Scales (B), from different geographical regions. In each panel, the distribution for one region is highlighted, and the distributions for the other regions are shown in grey. There are no Theory scales in the database from Oceania or Latin America (B).

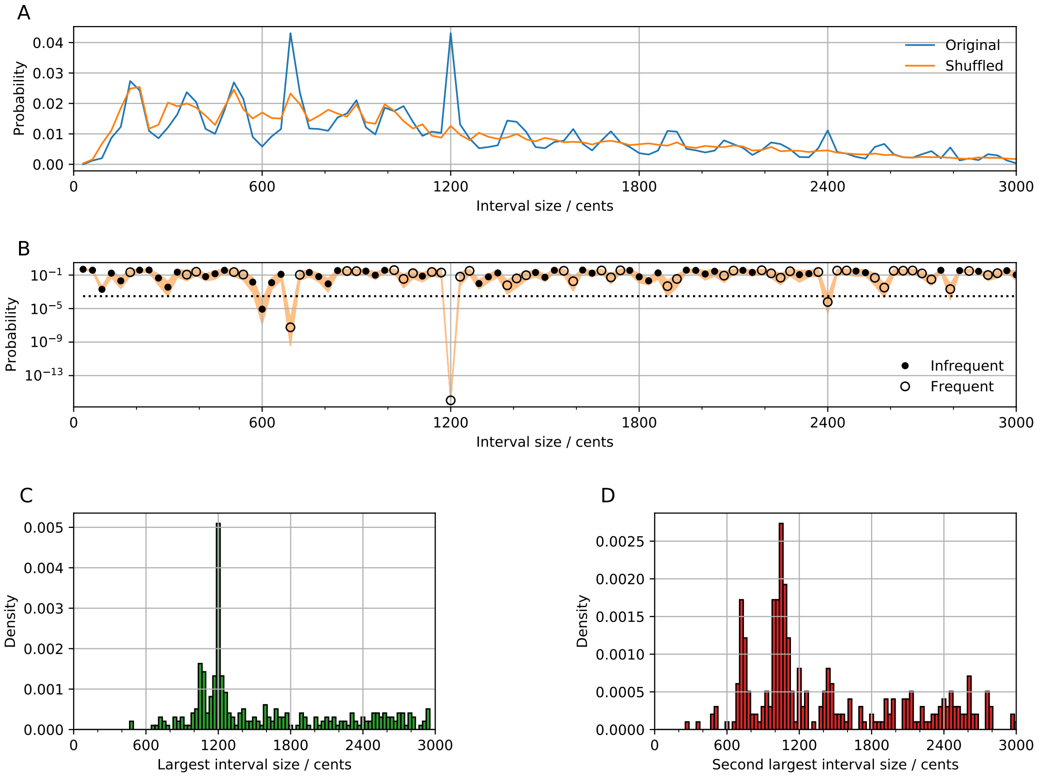

Figure 6: A: Distribution of notes in measured scales (blue; histogram bin size = 30 cents). Distribution of notes obtained via alternative sampling: shuffling the step sizes within scales (orange), for those scales that are far from equidistant (difference between minimum and maximum step sizes is greater than 100 cents). The x-axis is truncated at 3,000 cents for clarity. B: Probability that the counts observed in the original data were generated by the (far-from-equidistant) shuffled scales distribution.
The peak at 700 cents is lower than in the main text Fig. 3D. This indicates that for scales that are far from equidistant, the step sizes are arranged so that fifths appear more frequently than chance.

C: Distribution of the largest interval size for all scales. D: Distribution of the second largest interval size for all scales.
When we create new samples by shuffling without replacement, we do not count the final note since this will always be the same. We considered that if there were many equidistant scales, they would result in octave intervals appearing by chance. However, this does not happen as the second largest interval size falls short of an octave in many of these equidistant scales.

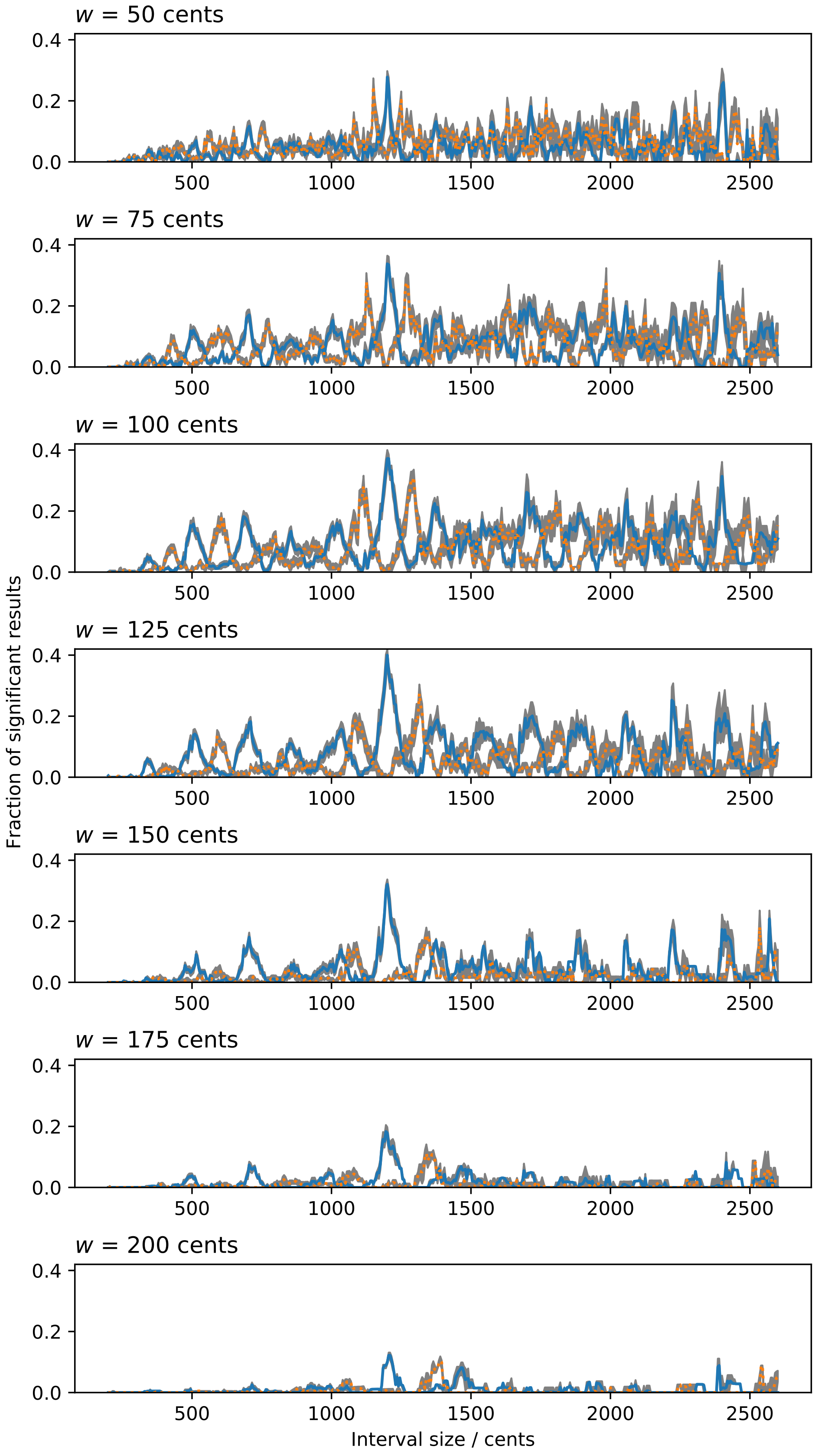

Figure 7: Effect of changing w on the fraction of significant results found for each interval size (see main text for details of statistical test). Blue line indicates that the interval is found significantly more than chance, while the orange line indicates the opposite. Shaded region shows bootstrapped 95% confidence intervals.

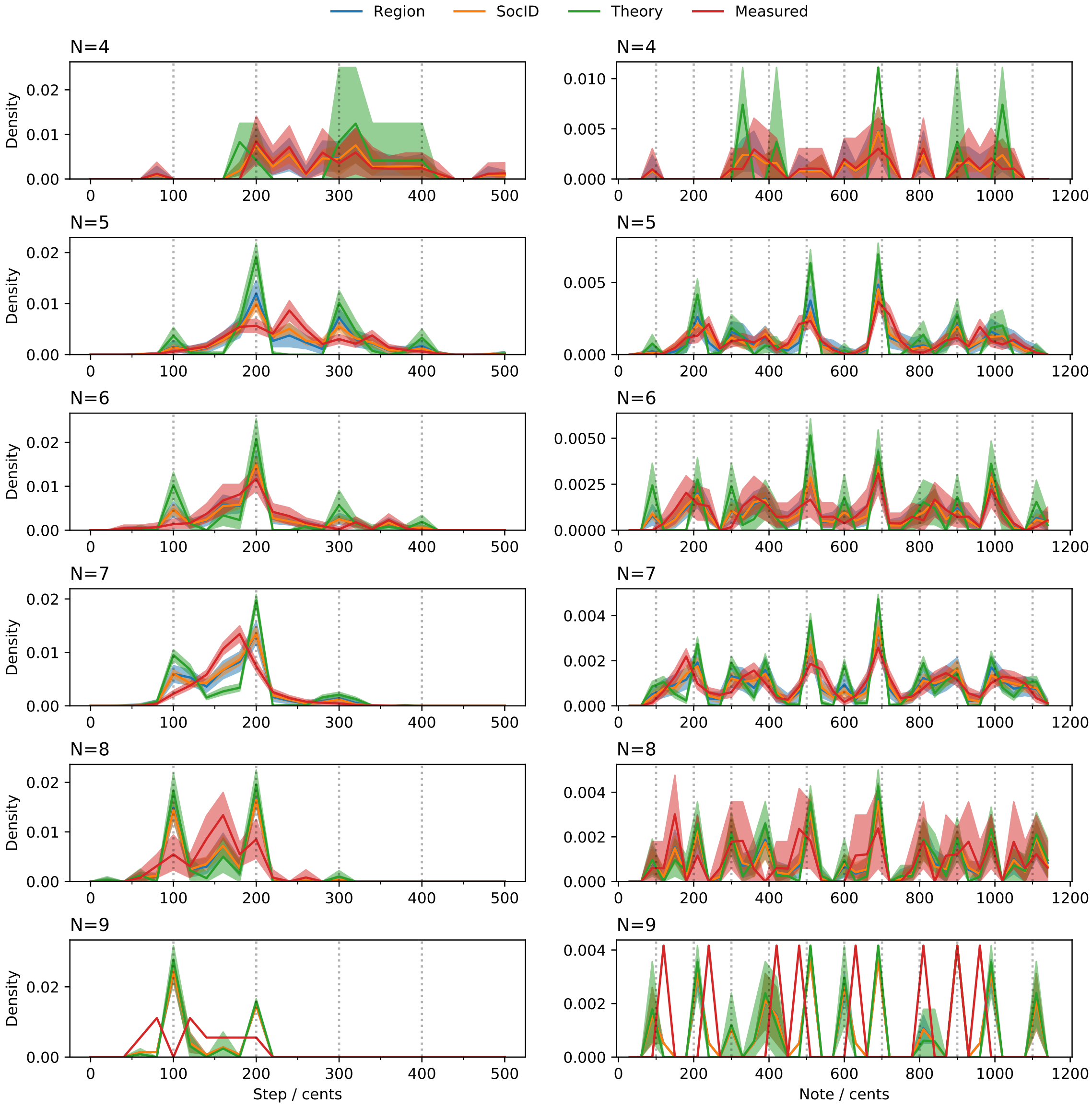

Figure 8: Step size and scale note distributions for the SocID subsample (max $N_{samp} = 5$), shown separately for sets of N-note scales. Separate lines are shown for alternative samples: Region (no more than 10 scales taken from each region); SocID (no more than 5 scales per society); only theory scales; only measured scales. Shaded region shows bootstrapped 95% confidence intervals.

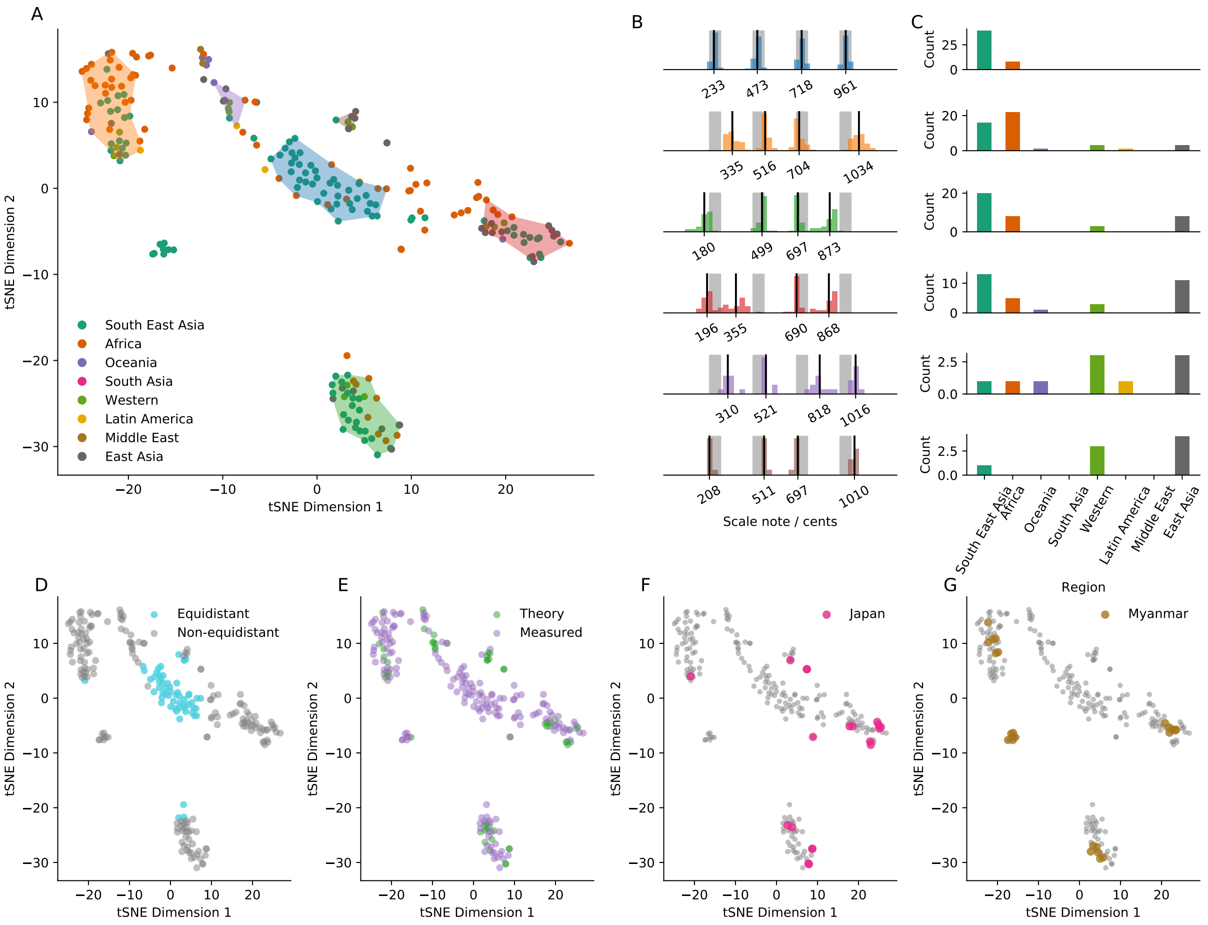

Figure 9: Cross-cultural diversity of scales. A: 2-dimensional embedding of 232 pentatonic scales, with the six largest clusters indicated (shaded areas). B: Note distributions for each cluster. Black lines are shown for means of each note, and grey shading indicates equidistant scale notes ±30 cents. C: Geographic distribution of each cluster. D-G: Embeddings are labelled with: equidistant scales (D; where notes are on average within 30 cents of the equidistant values), theory vs measured scales (E), Japanese scales (F), Burmese scales (G).

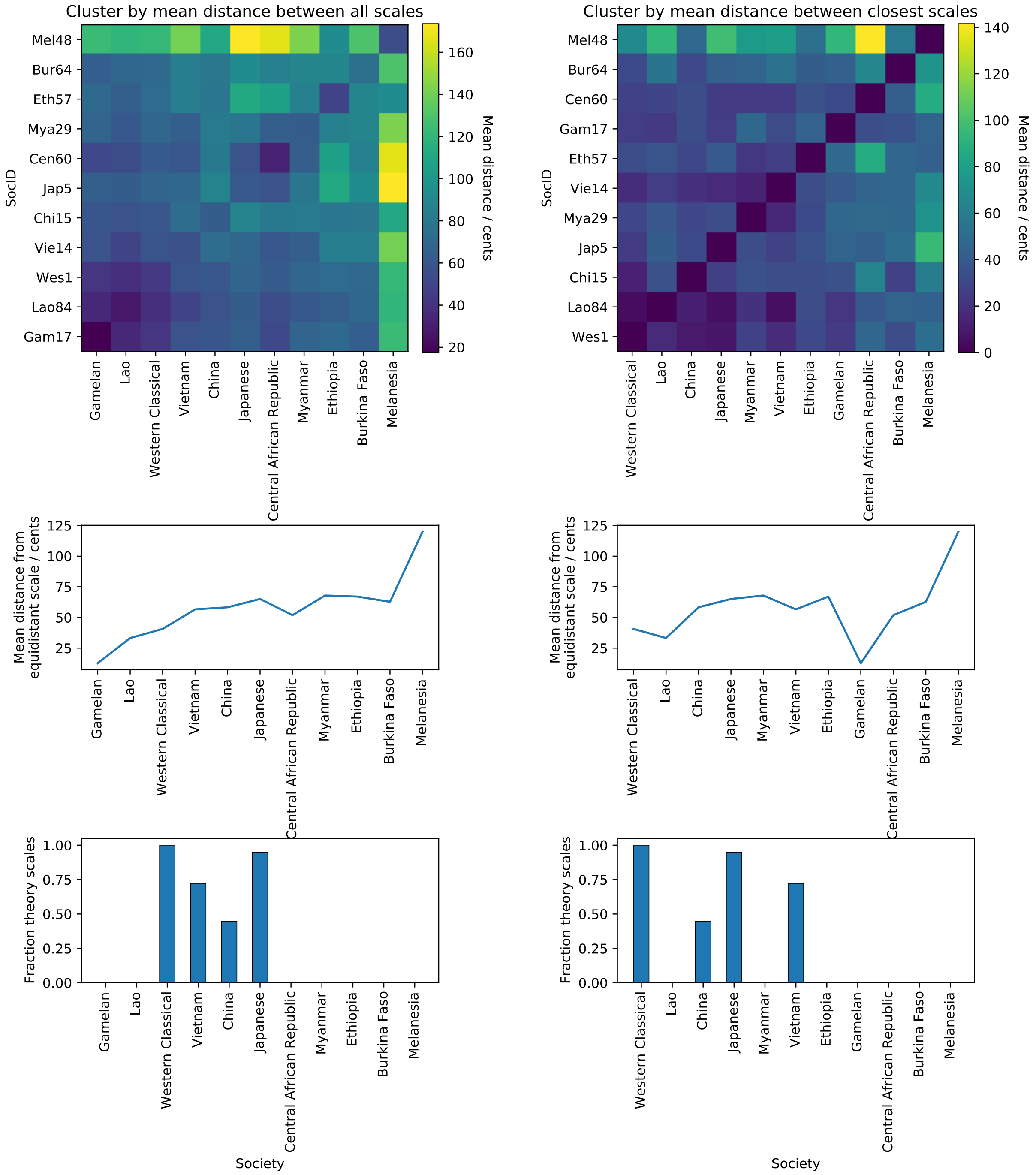

Figure 10: A: Societies clustered by distance between 5-note scales for two distance metrics: (Left) mean distance between all pairs of scales between two clusters (symmetric distance metric: distance from society A to society B is equal to the distance from society B to society A); clustering by the mean distance between all scales in society A, and the closest corresponding scale in society B (asymmetric distance metric). Societies are ordered according by hierarchical clustering according to each metric respectively, so that adjacent societies on the figure are more similar to each other. Societies are included in this analysis if there are at least 5 examples of scales. B: Mean distance from the equipentatonic scale. C: Fraction of scales found in the database that are theory scales.

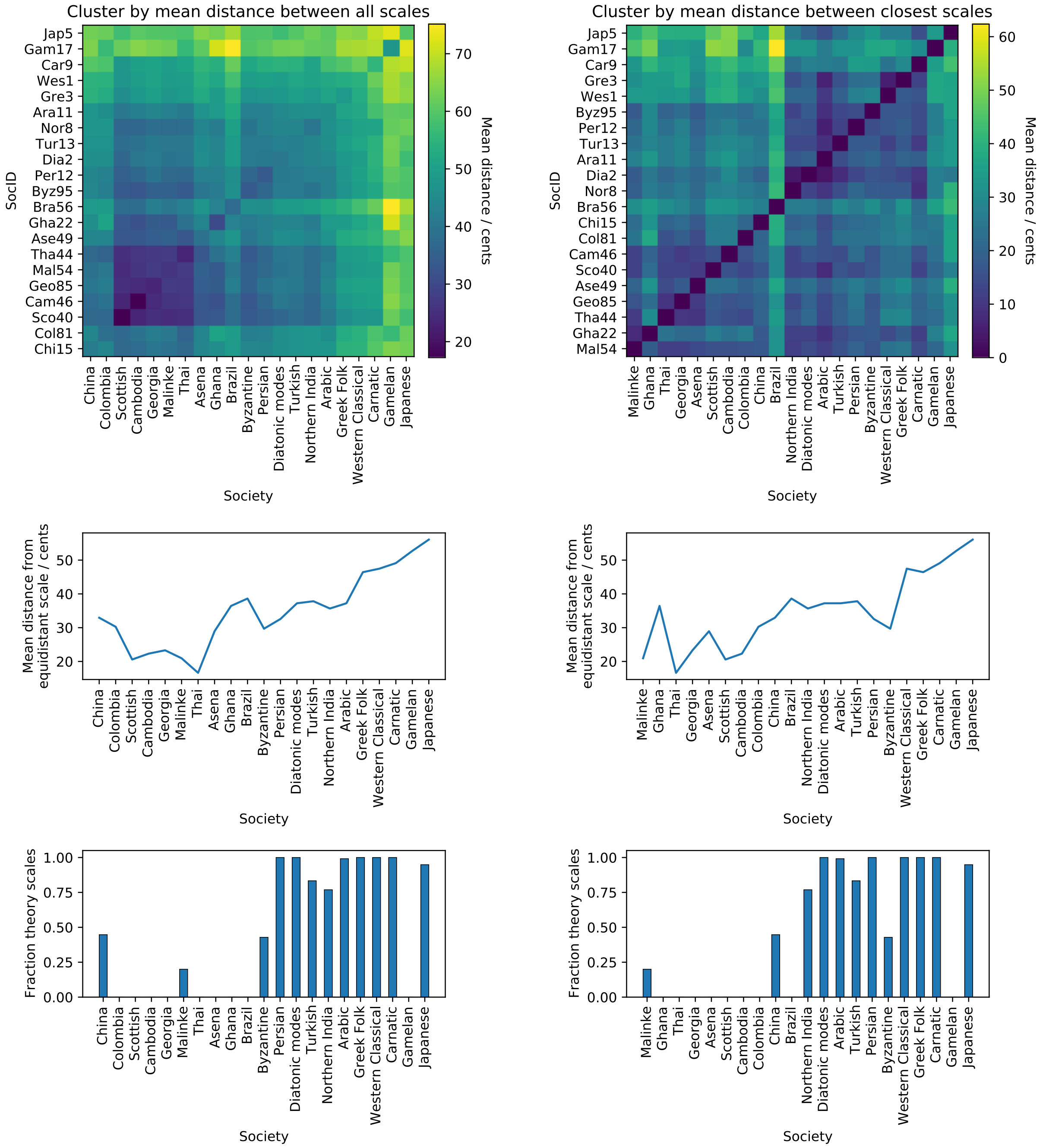

Figure 11: A: Societies clustered by distance between 7-note scales for two distance metrics: (Left) mean distance between all pairs of scales between two clusters (symmetric distance metric: distance from society A to society B is equal to the distance from society B to society A); clustering by the mean distance between all scales in society A, and the closest corresponding scale in society B (asymmetric distance metric). Societies are ordered according by hierarchical clustering according to each metric respectively, so that adjacent societies on the figure are more similar to each other. Societies are included in this analysis if there are at least 5 examples of scales. B: Mean distance from the equiheptatonic scale. C: Fraction of scales found in the database that are theory scales.

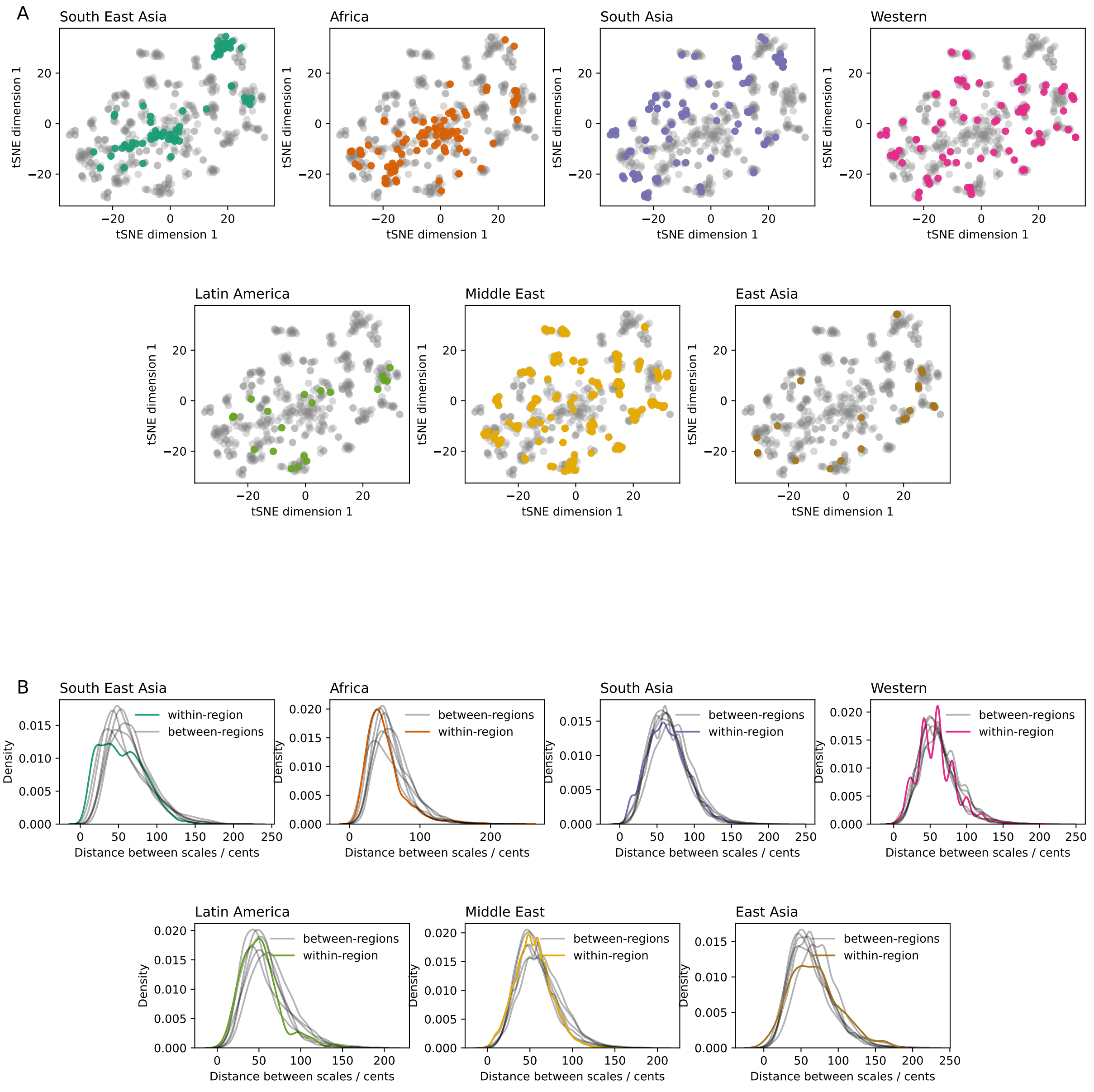

Figure 12: A: tSNE embedding of 7-note scales, with different regions highlighted in each plot. Oceania was not included since there are too few examples in this case. B: Distributions of distances between all possible pairs of 7-note scales between one region and another (between-region distance) is shown in grey; distributions of distances between all pairs of scales within a region (within-region distance) is highlighted in each plot.

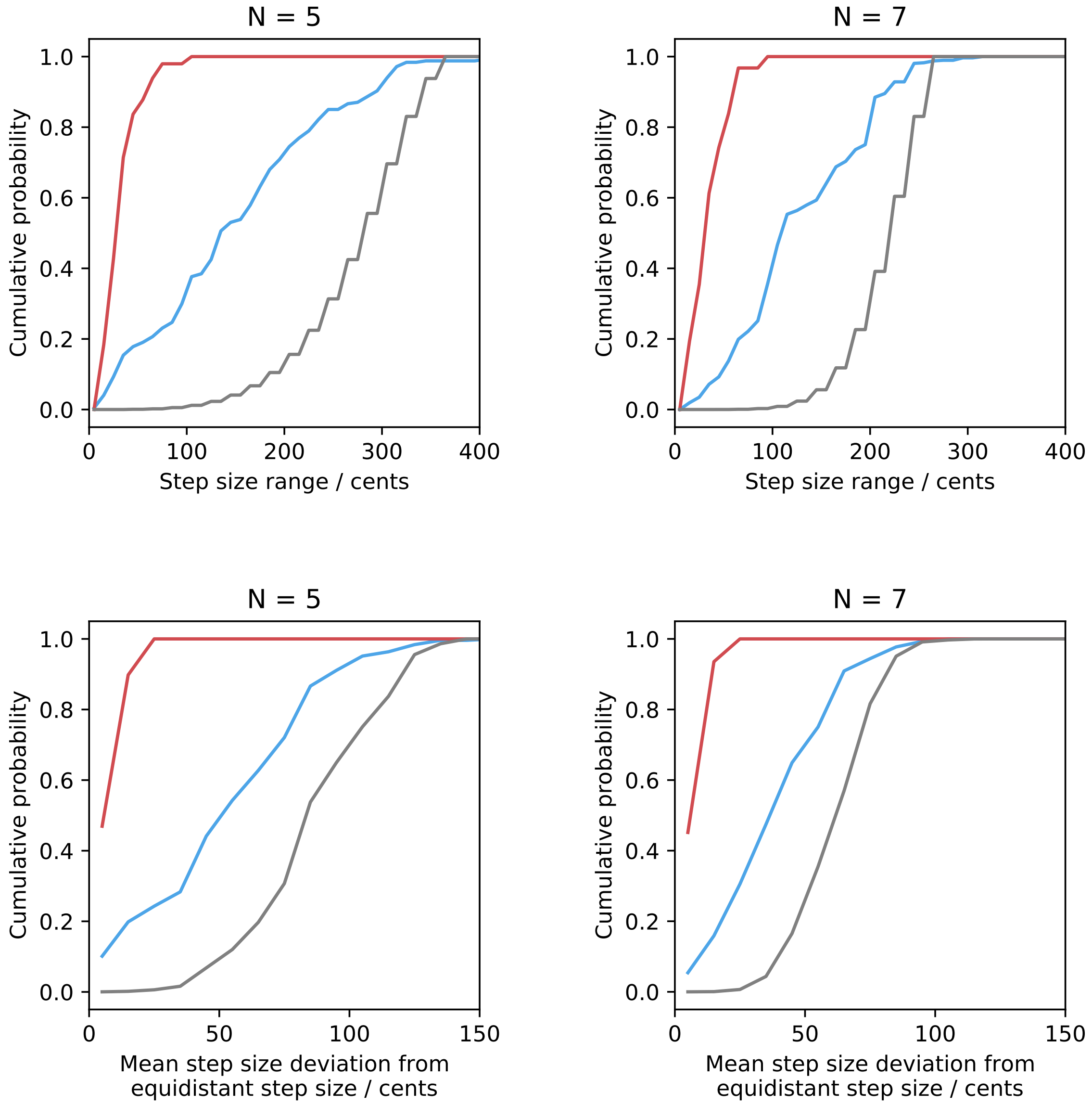

Figure 13: Distributions of alternative metrics of equidistance for real scales, grid scales, Gamelan slendro scales ($N = 5$), and Thai scales ($N = 7$). Top: difference between smallest and largest step size. Bottom: Mean deviation of step sizes from equidistant step size.

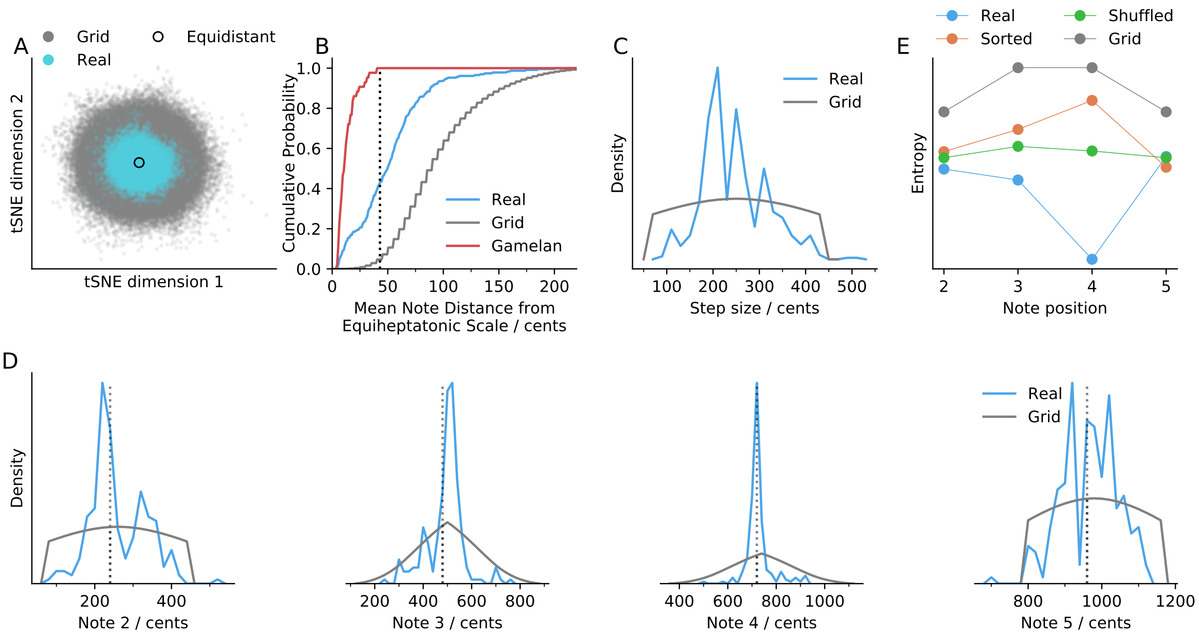

Figure 14: Comparison of real scales with all possible 5-note scales enumerated on a grid (20 cents resolution; with step sizes limited to 60-320 cents). A: Two-dimensional embedding of grid scales. Grid scales that correspond to real scales (notes are on average within 10 cents of a real scale) are highlighted cyan; a black circle shows the equidistant scale. B: Mean note distance from 5-note scales and the equipentatonic scale, for Thai scales, real scales, and all grid scales. C: Step size histograms (bin size = 20 cents) in real scales and grid scales. D: Scale note histograms (bin size = 20 cents) for notes 2-5 (no tonic and octave) for: real scales; real scales, but rearranged with their steps arranged in order of size (Sorted); real scales, but rearranged with their steps in random order (Shuffled); grid scales. E: Entropy of note distributions in D.

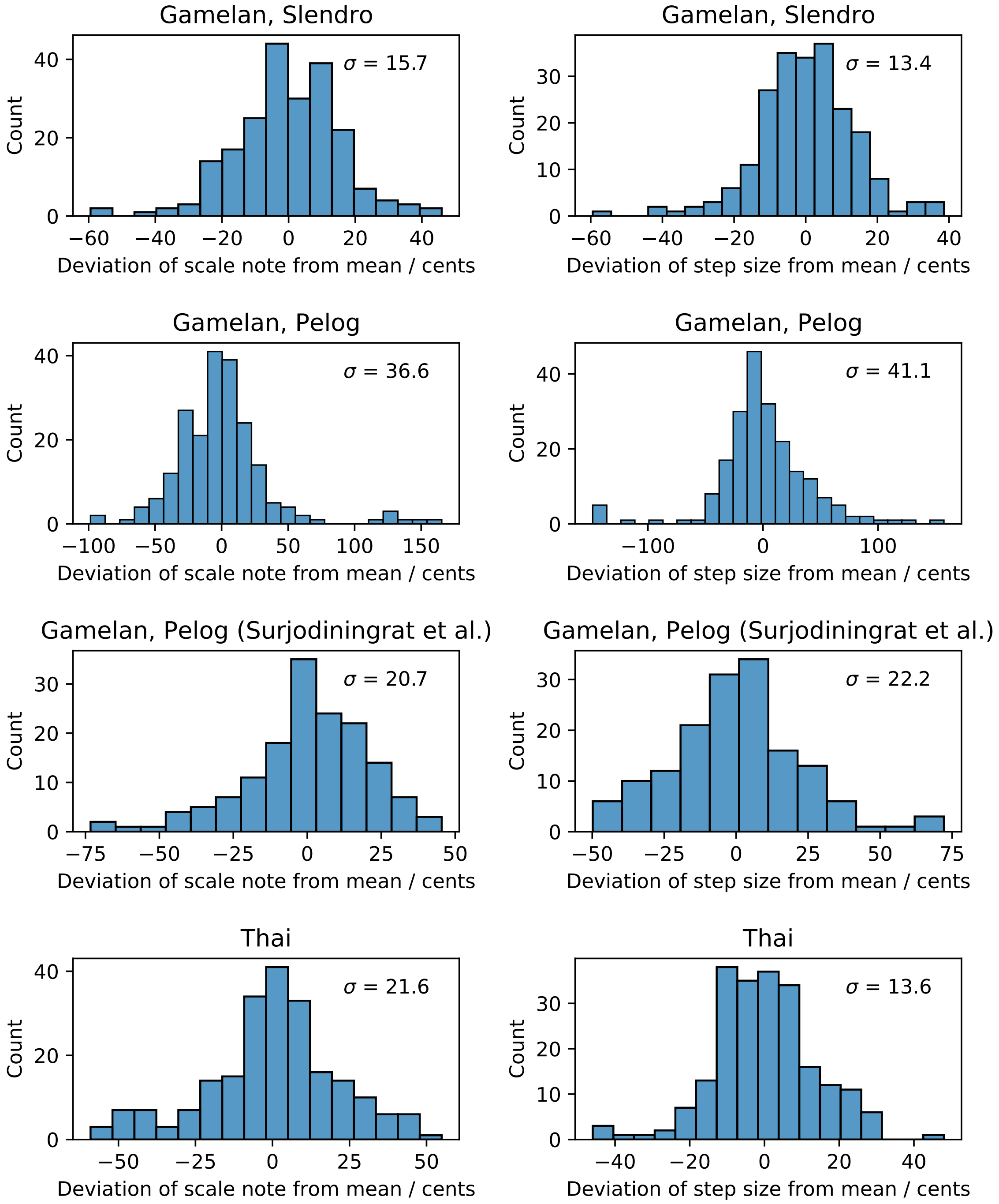

Figure 15: Deviation of scale notes and step sizes from their mean values, for scales in the database: Gamelan *slendro* scales (A), Gamelan *pelog* scales (B), Gamelan *pelog* scales from Surjodiningrat et al. [1] (C; this source accounts for 22 out of 30 *pelog* scales in the database), and Thai scales (D). Standard deviation is shown in text on each subplot.
We are not exactly sure why the variance of *pelog* scales is lower in Surjodiningra et al. than other sources, but it may have something to do with their attempt at only measuring tunings from 'outstanding' gamelan orchestras.

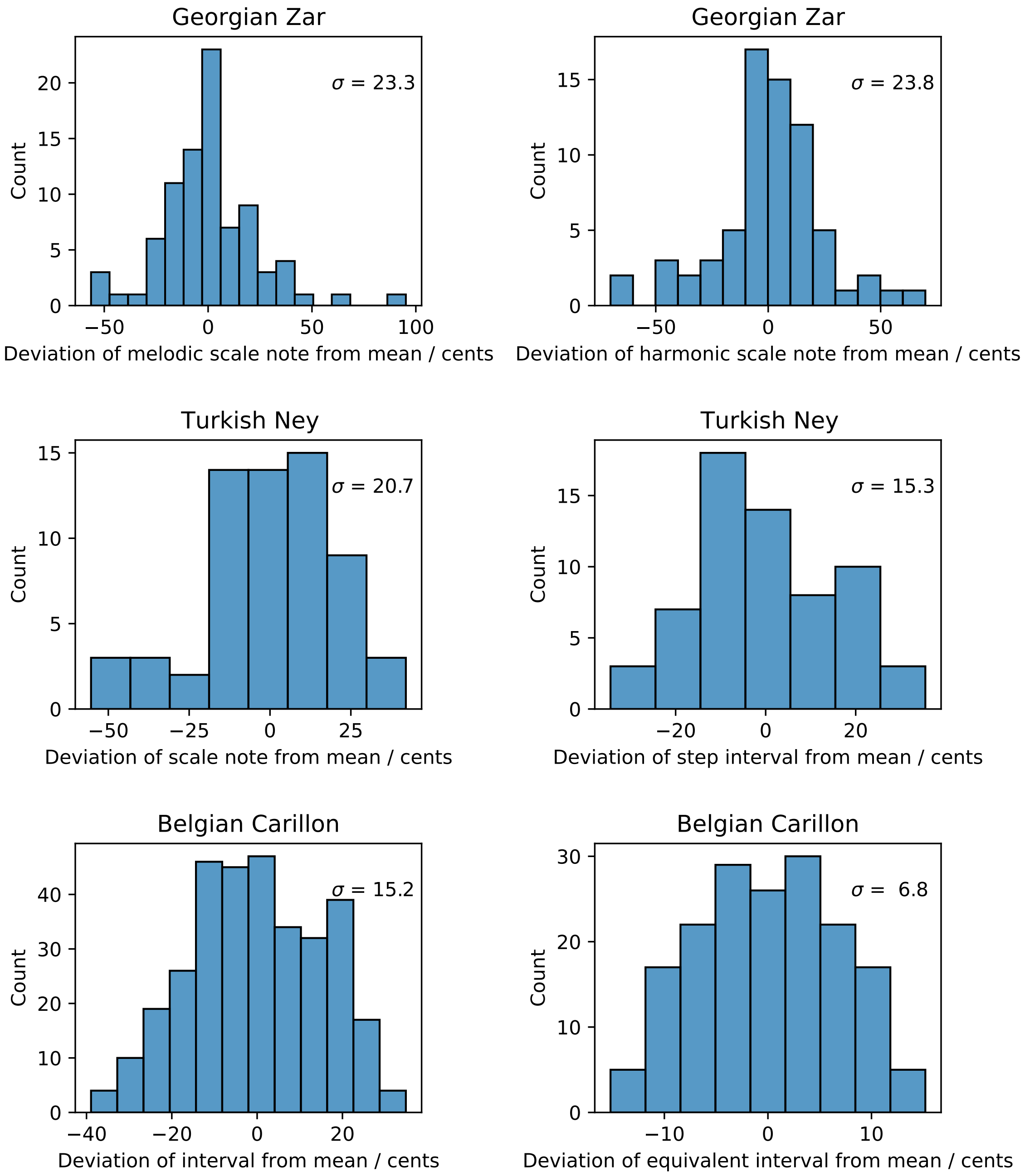

Figure 16: Analysis of interval consistency for tuning information from three sources: Georgian polyphonic singing (A), from recordings of Zar (a type of funeral dirge) taken in different villages; [2] a set of Turkish 'ney' flutes (B); [3] a single Belgian carillon. [4]

A: We align (in some villages certain notes are omitted) and compare all scale notes that were measured either from melodic pitch class histograms (left) or harmonic pitch class histograms (right).

B: Since the flute tunings are already aligned (same number of notes; similar scale notes), we can calculate the deviation from mean for each note position, for scale notes (left) and step sizes (right).

C: Unfortunately there is only one set of notes that spans 2,700 cents, so we performed two analyses of intervallic consistency within the instrument. We grouped all possible intervals by their distance to the nearest equal tempered (12-tet) interval, and calculated the deviation from the mean (left). It is not known whether the intended tuning is 12-tet (the authors speculate that mean-tempered tuning is used), so we also used a restricted set of intervals, where only intervals between the same pairs of notes were grouped together (right; *e.g.*, C3-D3 is only grouped with C4-D4). This results in most groups only having two sets of measurements, which artefactually results in a lower standard deviation. It can be shown that using a sample size of 2 to estimate the standard deviation will typically result in a value that is approximately half of the actual standard deviation; thus, we believe that the first measurement (C, left) is a good approximation of the interval consistency of this instrument.

Table 1: The number of scales of each type from each source, in order: raw theory; raw measured from instrument; raw measured from recording; octave theory; octave instrument; octave recording; total octave scales.

Ref	RefID	Year	T	M_{inst}	M_{rec}	OT	OM_{inst}	OM_{rec}	O_{total}
[5]	2	2018	239	54	3	192	49	3	244
[6]	1	2013	173	0	0	202	0	0	202
[1]	12	1972	0	51	0	0	50	0	50
[7]	16	1980	0	31	0	0	48	0	48
[8]	3	1885	25	29	0	21	22	0	43
[9]	40	2013	0	0	28	0	0	28	28
[10]	44	1975	0	3	0	0	24	0	24
[11]	51	1990	0	9	0	0	17	0	17
[12]	60	2013	8	0	8	6	0	8	14
[13]	34	2015	0	7	0	0	14	0	14
[14]	19	1982	14	0	0	13	0	0	13
[15]	54	1985	0	3	0	0	13	0	13
[16]	30	2005	0	4	0	0	11	0	11
[2]	56	2020	0	0	22	0	0	11	11
[17]	32	2009	0	4	0	0	9	0	9
[18]	13	1976	0	4	0	0	8	0	8
[19]	6	1967	0	16	0	0	8	0	8
[20]	17	1981	0	8	0	0	7	0	7
[3]	58	2011	0	0	9	0	0	7	7
[21]	35	2015	0	4	0	0	6	0	6
[22]	14	1979	0	9	0	0	6	0	6
[23]	8	1969	0	3	0	0	6	0	6
[24]	18	1982	0	2	0	0	6	0	6
[25]	23	1987	0	4	0	0	6	0	6
[26]	5	1964	0	5	0	0	6	0	6
[27]	24	1991	0	0	5	0	0	5	5
[28]	33	2010	0	4	1	0	4	1	5
[29]	15	1980	0	5	0	0	5	0	5
[30]	7	1967	0	3	8	0	0	5	5
[31]	46	1960	0	10	0	0	5	0	5
[32]	50	1978	0	5	0	0	5	0	5
[33]	29	2004	0	5	0	0	4	0	4
[34]	38	1976	0	0	4	0	0	4	4
[35]	37	2019	0	3	0	0	4	0	4
[36]	11	1976	0	4	0	0	4	0	4
[37]	31	2008	0	1	0	0	3	0	3
[38]	22	1986	0	4	0	0	3	0	3
[39]	39	1998	0	0	3	0	0	3	3
[40]	49	1992	0	1	0	0	3	0	3
[41]	59	2012	0	3	0	0	3	0	3
[42]	27	2001	0	2	0	0	2	0	2
[43]	26	1993	0	2	0	0	2	0	2
[44]	10	1971	0	2	0	0	2	0	2
[45]	9	1970	0	2	0	0	2	0	2
[46]	25	1991	0	1	0	0	2	0	2
[47]	4	1950	0	2	0	0	2	0	2
[48]	20	1984	0	1	0	0	2	0	2
[49]	21	1985	0	2	0	0	2	0	2
[50]	48	1963	0	1	0	0	2	0	2
[51]	52	1969	0	2	0	0	2	0	2
[52]	53	2018	0	0	2	0	0	2	2
[53]	36	2018	0	1	0	0	1	0	1
[54]	28	2004	0	2	0	0	1	0	1
[55]	45	1966	0	1	0	0	1	0	1
[56]	47	1962	0	2	0	0	1	0	1
[57]	55	2020	0	0	9	0	0	1	1
[58]	57	1937	0	1	0	0	1	0	1
[59]	41	2005	0	0	2	0	0	0	0
[60]	42	2006	0	0	7	0	0	0	0
[61]	43	1971	0	1	0	0	0	0	0
		**Total**	459	323	111	434	384	78	896

Table 2: The tunings used for ‘theory’ scales depending on culture.

Society	12-tet	24-tet	53-tet	Just Intonation	Pythagorean	Persian	Turkish	Shi-er-lu
Western Classical	X							
Jazz	X							
Diatonic modes	X			X	X			
Greek Folk	X							
Japanese					X			
Malinké					X			
Chinese								X
Hindustani	X			X				
Carnatic				X				
Arabian		X	X					
Persian						X		
Turkish			X				X	

References

[1] W. Surjodiningrat, A. Susanto, and P. J. Sudarjana. *Tone Measurements of Outstanding Javanese Gamelans in Jogjakarta and Surakarta.* Gadjah Mada University Press, 1972.

[2] N. Mzhavanadze and F. Scherbaum. Svan funeral dirges (zär): Musicological analysis. *Musicologist*, 4(2):168–197, 2020. doi: 10.33906/musicologist.782185.

[3] A. Tan. Ney açkısının tarihi ve teknik gelişimi. *Yayınlanmamış Doktora Tezi. Marmara Üniversitesi, Sosyal Bilimler Enstitüsü, İstanbul*, 2011.

[4] A. Schneider and M. Leman. *Sound, Pitches and Tuning of a Historic Carillon*, pages 247–298. Springer International Publishing, Cham, 2017. doi: 10.1007/978-3-319-47292-8_9.

[5] H. Rechberger. *Scales and Modes Around the World: The Complete Guide to the Scales and Modes of the World.* Fennica Gehrman Ltd., 2018.

[6] M. J. Hewitt. *Musical Scales of the World.* Note Tree, 2013.

[7] W. Van Zanten. The equidistant heptatonic scale of the asena in malawi. *Afr. Music*, 6(1):107–125, 1980. doi: 10.21504/amj.v6i1.1099.

[8] A. J. Ellis. *On the Musical Scales of Various Nations.* Journal of the Society of arts, 1885.

[9] S. Weisser and F. Falceto. Investigating qanat in amhara secular music: An acoustic and historical study. *Annales d'Éthiopie*, 28(1):299–322, 2013. doi: 10.3406/ethio.2013.1539.

[10] Robert Garfias. Preliminary thoughts on burmese modes. *Asian Music*, 7(1):39–49, 1975.

[11] C. Miñana Blasco. Afinación de las marimbas en la costa pacífica colombiana: Un ejemplo de la memoria interválica africana en colombia. 1990.

[12] Maria Panteli and Hendrik Purwins. A quantitative comparison of chrysanthine theory and performance practice of scale tuning, steps, and prominence of the octoechos in byzantine chant. *J. New Music Res.*, 42(3):205–221, 2013. doi: 10.1080/09298215.2013.827215.

[13] J. Garzoli. The myth of equidistance in thai tuning. *Anal Approaches Music*, 4(2):1–29, 2015.

[14] Ho Lu-Ting and Han Kuo-huang. On chinese scales and national modes. *Asian Music*, 14(1):132–154, 1982. doi: 10.2307/834047.

[15] T. E. Miller. *Traditional Music of the Lao: Kaen Playing and Mawlum Singing in Northeast Thailand.* Number 13. Praeger, 1985.

[16] W. A. Sethares. *Tuning, Timbre, Spectrum, Scale.* Springer Science & Business Media, 2005.

[17] J. L. Strand. *The Sambla Xylophone: Tradition and Identity in Burkina Faso.* PhD thesis, Wesleyan University, Connecticut, 2009.

[18] D. Morton and C. Duriyanga. *The Traditional Music of Thailand*, volume 8. Univ of California Press, 1976.

[19] R. Brandel. *The Music of Central Africa: An Ethnomusicological Study: Former French Equatorial Africa the Former Belgian Congo, Ruanda-Urundi Uganda, Tanganyika.* Springer Science & Business Media, 1967.

[20] Hugo Zemp. Melanesian solo polyphonic panpipe music. *Ethnomusicology*, 25(3):383–418, 1981. doi: 10.2307/851551.

[21] N. Wisuttipat. Relative nature of thai traditional music through its tuning system. *International Journal of Creative and Arts Studies*, 2(1):86–97, 2015. doi: 10.24821/ijcas.v2i1.1441.

[22] Joerg Haeberli. Twelve nasca panpipes: A study. *Ethnomusicology*, 23(1):57–74, 1979. doi: 10.2307/851338.

[23] Gilbert Rouget and J. Schwarz. Sur les xylophones équiheptaphoniques des malinké. *Rev. Musicol.*, 55(1):47–77, 1969. doi: 10.2307/927751.

[24] B. A. Aning. Tuning the kora: A case study of the norms of a gambian musician. *J. Afr. Stud.*, 9(3):164, 1982.

[25] R. Yu-An, E. C. Carterette, and W. Yu-Kui. A comparison of the musical scales of the ancient chinese bronze bell ensemble and the modern bamboo flute. *Percept. Psychophys.*, 41(6):547–562, 1987. doi: 10.3758/BF03210489.

[26] Gerhard Kubik. Harp music of the azande and related peoples in the central african republic: (part i - horizontal harp playing). *Afr. Music*, 3(3):37–76, 1964.

[27] D. H. Keefe, E. M. Burns, and P. Nguyen. Vietnamese modal scales of the dan tranh. *Music Percept.*, 8(4):449–468, 1991. doi: 10.2307/40285522.

[28] M. Kuss. *Music in Latin America and the Caribbean: An Encyclopedic History: Volume 1: Performing Beliefs: Indigenous Peoples of South America, Central America, and Mexico.* University of Texas Press, 2010.

[29] Gerhard Kubik. Likembe tunings of kufuna kandonga (angola). *Afr. Music*, 6(1):70–88, 1980.

[30] J. Kunst. *Music in New Guinea.* Brill, 1967.

[31] A. M. Jones. Indonesia and africa: The xylophone as a culture-indicator. *African Music : Journal of the International Library of African Music*, 2(3):36–47, 1960. doi: https://doi.org/10.21504/amj.v2i3.608.

[32] Alan Thrasher. The transverse flute in traditional chinese music. *Asian Music*, 10(1):92–114, 1978.

[33] J. Zhang, X. Xiao, and Y. K. Lee. The early development of music. analysis of the jiahu bone flutes. *Antiquity*, 78 (302):769–778, 2004. doi: 10.1017/S0003598X00113432.

[34] C. M. L. Kimberlin. *Masinqo and the Nature of Qanat.* PhD thesis, The University of California, Los Angeles, 1976.

[35] R. Bader. Temperament in tuning systems of southeast asia and ancient india. In *Computational Phonogram Archiving*, pages 75–107. Springer, 2019. doi: 10.1007/978-3-030-02695-0_3.

[36] R. Knight. Kora music from the gambia, played by foday musa suso, 1976.

[37] L. E. McNeil and S. Mitran. Vibrational frequencies and tuning of the african mbira. *J. Acoust. Soc. Am.*, 123(2): 1169–1178, 2008. doi: 10.1121/1.2828063.

[38] Robert Gottlieb. Sudan ii: Music of the blue nile province; the ingessana and berta tribes, 1986.

[39] Unjung Nam. Pitch distributions in korean court music: Evidence consistent with tonal hierarchies. *Music Percept.*, 16(2):243–247, 1998. doi: 10.2307/40285789.

[40] Gerhard Kubik. Embaire xylophone music of samusiri babalanda (uganda 1968). *The World of Music*, 34(1):57–84, 1992.

[41] M. Mendes et al. *Fé No Pife: As Flautas De Pífano No Contexto Cultural Da Banda Cabaçal Dos Irmãos Aniceto.* PhD thesis, Universidade do Estado de Santa Catarina, 2012.

[42] Albrecht Schneider. Sound, pitch, and scale: From ”tone measurements” to sonological analysis in ethnomusicology. *Ethnomusicology*, 45(3):489–519, 2001. doi: 10.2307/852868.

[43] Edward C. Carterette, Roger A. Kendall, and Sue Carole De Vale. Comparative acoustical and psychoacoustical analyses of gamelan instrument tones. *Journal of the Acoustical Society of Japan (E)*, 14(6):383–396, 1993. doi: 10.1250/ast.14.383.

[44] A. Tracey. The nyanga panpipe dance. *Afr. Music*, 5(1):73–89, 1971. doi: 10.21504/amj.v5i1.1152.

[45] A. Tracey. The matepe mbira music of rhodesia. *Afr. Music*, 4(4):37–61, 1970. doi: 10.21504/amj.v4i4.1681.

[46] A. Tracey. Kambazithe makolekole and his valimba group: A glimpse of the technique of the sena xylophone. *Afr. Music*, 7(1):82–104, 1991. doi: 10.21504/amj.v7i1.1932.

[47] K. P. Wachsmann. An equal-stepped tuning in a ganda harp. *Nature*, 165(4184):40–41, 1950. doi: 10.1038/165040a0.

[48] G. Kubik. A structural examination of homophonic multi-part singing in east and central africa. *Anuario Musical*, 39:27, 1984.

[49] Gerhard Kubik. African tone-systems: A reassessment. *Yearb. Tradit. Music*, 17:31–63, 1985. doi: 10.2307/768436.

[50] G. Kubik. Discovery of a trough xylophone in northern mozambique. *African Music : Journal of the International Library of African Music*, 3(2):11–14, 1963. doi: https://doi.org/10.21504/amj.v3i2.826.

[51] J. Sundberg and P. Tjernlund. Computer measurements of the tone scale in performed music by means of frequency histograms. *STL-QPS*, 10(2-3):33–35, 1969.

[52] Lyndsey Copeland. Pitch and tuning in beninese brass bands. *Ethnomusicology Forum*, 27(2):213–240, 2018. doi: 10.1080/17411912.2018.1518151.

[53] A. Morkonr, S. Punkubutra, et al. The collecting process of xylophone's sound d (ranād xek) from art to numerical data. In *2018 International Conference on Engineering, Applied Sciences, and Technology (ICEAST)*, pages 1–4, 2018. doi: 10.1109/ICEAST.2018.8434434.

[54] K. Attakitmongcol, R. Chinvejkitvanich, and S. Sujitjorn. Characterization of traditional thai musical scale. In *Proceedings of the 5th WSEAS International Conference on Acoustics and Music: Theory & Applications (AMTA'04)*, 2004.

[55] A. M. Jones. A kwaikèr indian xylophone. *Ethnomusicology*, 10(1):43–47, 1966.

[56] G. Kubik. The endara xylophone of bukonjo. *African Music : Journal of the International Library of African Music*, 3(1):43–48, 1962. doi: https://doi.org/10.21504/amj.v3i1.736.

[57] Frank Scherbaum, Nana Mzhavanadze, Simha Arom, Sebastian Rosenzweig, and Meinard Müller. *Tonal Organization of the Erkomaishvili Dataset: Pitches, Scales, Melodies and Harmonies.* Number 1. 2020. doi: 10.25932/publishup-47614.

[58] M. Mokhtar and A. M. Mosharrafa. Modes in modern egyptian music. *Nature*, 140(3543):548–549, 1937. doi: 10.1038/140548b0.

[59] Rytis Ambrazevičius. The perception and transcription of the scale reconsidered: Several lithuanian cases. *The World of Music*, 47(2):31–53, 2005.

[60] Rytis Ambrazevičius. Modelling of scales in traditional solo singing. *Music. Sci.*, 10(1_suppl):65–87, 2006. doi: 10.1177/1029864906010001041.

[61] P. R. Cooke. Ludaya - a transverse flute from eastern uganda. *Yearb. Int. Folk Music Council*, 3:79–90, 1971. doi: 10.2307/767457.
