# Supplementary material for: Convergent evolution in a large cross-cultural database of musical scales
Source: PLoS One. 2023 Dec 13;18(12):e0284851. doi: 10.1371/journal.pone.0284851 (PMC10718441; doi:10.1371/journal.pone.0284851)
Supplement: Attachment — Submitted filename: PlosOneResponse_final.pdf [file pone.0284851.s003.pdf]

Reviewer #1: The revision addresses many of the reviewers' comments and the remaining disagreements are no more than normal for scientific discourse.

I note that a recently published paper (Anglada-Tort, M., Harrison, P. M., Lee, H., & Jacoby, N. (2023). Large-scale iterated singing experiments reveal oral transmission mechanisms underlying music evolution. Current Biology) directly addresses material in this submission,focally but not limited to discussion on lines 472ff.

Response: This comment was addressed in the previous revision. See reference [160], and the two mentions of it in the text.

Reviewer #2: I appreciate the attention to detail and care that the authors took in addressing the reviewer comments, and I think the paper is much clearer in its current form.

I will add two minor points, one to clarify a point from my first review, and another on one aspect that is still unclear to me:

1. "The relationship between melodic scales and harmony was not discussed. Conceivably harmony is a constraint on scale systems as well?"
- There is a world in which people would use notes within a harmonic context but not use those same notes within melodies, but to my knowledge that doesn't appear to happen. So it does seem like aspects like fusion/harmony constrain (or maybe, better put, guide) individuals to use particular notes in melodies. The added comments about fusion maybe address this issue sufficiently; I'll leave that to your discretion. I hope this clarifies my point.

Response: This is a good comment (first two sentences) by the reviewer, and was originally in an earlier version of the manuscript, and I had even done some research on whether there are counter examples (I have not found any yet). I removed it due to the need to shorten the paper. The section "How are scales selected?" does treat with harmony through both tonal fusion, sensory dissonance, and nuerodynamic theory.

2. The bar labels are still unclear to me on Figure 2. I only see one country labeled in Latin America, and many South America. Why is the label 'Latin America'?

Response: "Latin America" was used instead of Central America or South America, since I have few examples from either area. I grouped them together just for simplification (since there are few examples, it doesn't substantially affect any analysis). Just to clarify, 'Latin America' is a cultural term rather than geographic, and describes the countries in Central and South America that speak languages derived from Latin.
